# The effect of heated tobacco products on metabolic syndrome: A cohort study

**DOI:** 10.18332/tid/194490

**Published:** 2024-12-16

**Authors:** Yongho Jee, Sang Yop Shin, Mikyung Ryu, Jonathan M. Samet

**Affiliations:** 1 Advanced Biomedical Research Institute, Ewha Womans University Seoul Hospital, Seoul, Republic of Korea; 2 Korea Medical Institute, Seoul, Republic of Korea; 3 Institute on Aging, Ajou University Medical Center, Suwon, Republic of Korea; 4 Colorado School of Public Health, University of Colorado, Aurora, United States

**Keywords:** non-cigarette tobacco products, prevention, smoking caused disease

## Abstract

**INTRODUCTION:**

Evidence concerning the health effects of using heated tobacco products is needed. The aim of the present study is to investigate the effects of conventional cigarettes (CCs), electronic vaping cigarettes (EVCs), and heated tobacco products (HTPs) on the development of metabolic syndrome.

**METHODS:**

We conducted a cohort study in South Korea using data from the Korea Medical Institute. The study included 183870 people who visited the Korea Medical Institute, responded to a smoking status questionnaire in 2019, and were followed up in 2020 – ever and current use of CCs, EVCs, and HTPs. We defined the main outcome as incident metabolic syndrome among participants with at least two health checkups separated by a follow-up period of at least a year.

**RESULTS:**

The association of using HTPs with risk for metabolic syndrome was estimated after controlling for age, sex, exercise, drinking history, and smoking regular cigarettes and using EVCs, with the Cox proportional hazards model. The risk of incident metabolic syndrome was increased by 68% (HR=1.68; 95% CI: 1.25–2.26) for current HTP users compared to never users. Among HTP users who did not currently smoke conventional cigarettes, the risk was doubled (HR=2.17; 95% CI: 1.31–3.62) when their smoking duration was ≥3 years. The risk of metabolic syndrome increased by 33% (HR=1.33; CI: 1.18–1.49) among HTP users who used them more than 16 times a day. The use of HTP was found to increase the risk of developing metabolic syndrome, with a particularly elevated risk observed among those who used HTPs for more than three years. The risk was higher than that observed in conventional cigarette users.

**CONCLUSIONS:**

Our findings indicate that HTP use poses comparable risks in relation to metabolic syndrome development.

## INTRODUCTION

Over the last 15 years, major tobacco companies have developed and marketed nicotine-containing products, including electronic cigarettes (e-cigarettes) and heated tobacco products (HTPs) devices that deliver a nicotine-containing vapor generated without combustion^1^. The products initially marketed were vaping devices, which we refer to as electronic vaping cigarettes (EVCs). More recently, HTPs have been introduced into the marketplace in several regions^2-4^. The HTP devices heat a disposable tobacco stick with a thin metallic blade, maintaining the stick at a controlled temperature of up to 350°C. The heat creates the nicotine-containing vapor inhaled by the user without combustion and production of ash or smoke. By contrast, the EVCs aerosolize a liquid containing flavorings, propylene glycol, and vegetable glycerol^5^.

In terms of public health, a major question concerning the entry of HTPs into the marketplace is the extent to which the health risks they pose are reduced compared to the risks of conventional cigarettes and EVCs. Tobacco companies have increasingly marketed heated tobacco products (HTPs) under the guise of providing a healthier alternative to traditional smoking. However, studies show that these marketing strategies are similar to those used for slim and additive-free cigarettes, which falsely convey a reduced health risk. This has led to a proliferation of HTP use, particularly driven by misconceptions of health benefits. As previous research indicates, marketing HTPs as ‘reduced risk’ products may mirror the same tactics used for ‘light’ and ‘mild’ cigarettes, which were eventually banned for their misleading health claims^6^; with only a short time interval since the emergence of HTP, evidence on their risks is quite limited^1,7-14^. To date, most reports related to HTP address the toxic components of the aerosol and the associated risks, and epidemiological studies are lacking^1,4,8^. At the least, HTPs maintain nicotine addiction^1^.

Even as the prevalence of HTP use is increasing^14^, research on the short-term or long-term health effects of e-cigarettes is insufficient and lagging behind their increased usage. Here, we report the findings of a cohort study in Korea on the use of tobacco products, including HTPs, and the risk of developing metabolic syndrome. This syndrome is generally defined as comprising at least three of the following: abdominal obesity, high blood pressure, high blood sugar, high serum triglycerides, and low serum high-density lipoprotein (HDL)^15^. Metabolic syndrome is a substantial contributor to the global disease burden^16^. Research has linked smoking conventional cigarettes^17^ and the use of EVCs^18^ to metabolic syndrome, providing a rationale for investigating HTPs as well. Since many users of HTPs also smoke cigarettes and use EVCs^19^, the risks of this mixed exposure pattern also need to be investigated.

The periodic health checkups carried out in Korea provided the opportunity to carry out a cohort study on HTPs and risk for metabolic syndrome. We investigated the risk of metabolic syndrome incidents among people who visited the Korea Medical Institute for health checkups in 2018. This longitudinal cohort study aims to investigate the effects of conventional cigarettes, EVCs, and HTPs on the development of metabolic syndrome.

## METHODS

### Data source, study sample, and study design

**Figure 1:**
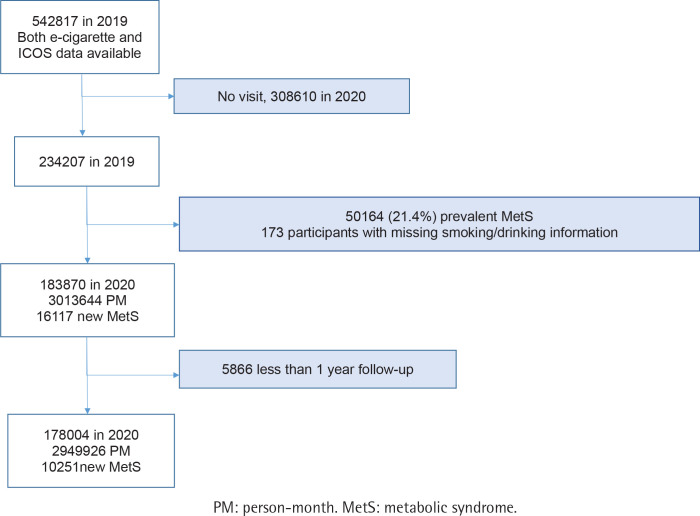
Data collection framework, The Korea Medical Institute study

A study cohort was created from participants in a health checkup in 2019 when the routine questionnaire first included HTP use (Figure 1). Of the total of 542817 potential participants, 50164 with metabolic syndrome in 2019 were excluded, along with those lacking smoking and alcohol data. Participants were monitored from 2019 until their examination in 2020, resulting in a final sample of 178004, all of whom had at least one year of follow-up (Figure 1). Thus, follow-up began with the baseline questionnaire in 2019 and ended at the time of the follow-up examination in 2020. Our study’s data usage and study design were approved by the Institutional Review Board of Ewha Women’s University Seoul Hospital, and informed consent was obtained from each subject (SEUMC 2021-07-007-001).

### Data collection

In Korea, questions on EVC and HTP use have been included in the general health checkup questionnaire of the National Health Insurance System (NHIS) since 2019. Data on tobacco product use are collected by self-report with the following questions: 1) ‘Have you ever used conventional cigarettes, electronic vaping cigarettes (EVCs), heated tobacco products (HTPs) in your lifetime?’; and 2) ‘Have you used conventional cigarettes, electronic vaping cigarettes (EVCs), heated tobacco products (HTPs) within the past 30 days?’. Participants who responded ‘yes’ to the first question and ‘no’ to the second question were categorized as ever conventional cigarette (CC), electronic cigarette (e-cigarette), and HTP users. Participants who responded ‘yes’ to both questions were categorized as current conventional cigarette/electronic cigarette (e-cigarette) and HTP users. Additionally, those who were categorized as current conventional cigarette/electronic vaping cigarette (EVC)/heated tobacco product (HTP) users, were asked to report the amount of use as follows: ‘How many cigarettes did you smoke per day on average? (CCs)’; ‘Have you ever used e-cigarettes during last month (EVCs)’/‘How long did you use heated tobacco products (HTPs)?’ (Supplementary file Material 1).

Using the World Health Organization classification, a current smoker was defined as someone who had smoked more than 100 cigarettes in their lifetime and smoked currently; a former smoker as someone who had smoked more than 100 cigarettes in the past and did not smoke currently; and a never smoker was defined as anyone who had never smoked more than 100 cigarettes and did not smoke currently^18^.

Metabolic syndrome was defined based on the modified Third National Cholesterol Education Program Expert Panel on Detection, Evaluation, and Treatment of High Blood Cholesterol in Adults (NCEP-ATP III) criteria^20^ along with the abdominal obesity criteria from the Korean Society for the Study of Obesity^21^. Metabolic syndrome was diagnosed when three or more of the following criteria were met: 1) waist circumference (WC) of ≥90 cm in men and ≥85 cm in women, 2) triglyceride (TG) concentration of ≥150 mg/dL, 3) HDL-cholesterol concentration of <40 mg/dL in men and <50 mg/dL in women; and 4) blood pressure (BP) ≥130/85 mmHg^18^.

### Outcome

The main outcome was incident metabolic syndrome among participants who revisited for a health checkup within two years and were diagnosed with metabolic syndrome during the checkup. We classified the participants by the number of components of metabolic syndrome present at baseline, ranging from 0 to 2. Over follow-up, incident metabolic syndrome was defined when the total number of components reached three over at least one year of follow-up. Participants were stratified based on the use of HTP. Details on the change in metabolic components between 2019 and 2020 were stratified by tobacco products and are summarized in the Supplementary file Material 2.

### Statistical analysis

Comparisons between tobacco product use groups were performed using a one-way analysis of variance for normally distributed continuous variables and chi-squared tests for categorical variables. Duration of HTP use was classified as: 1, 2, and ≥3 years. The frequency of HTP use was categorized as: 1–5, 6–10, 11–15, and ≥16 times per day. The Cox proportional hazard model was used to estimate the risk of metabolic syndrome associated with HTP use, with adjustment for age, sex (male/female), alcohol drinking status (number of drinks per week: 0, 1–5, ≥6), and exercise (days of high-intensity exercise per week: 0, 1–2, 3–4, ≥5), conventional cigarette use, and e-cigarette use.

The proportional hazard assumption was tested by using Schoenfeld residuals. Survival curves by current HTP status were plotted using the life table method. In addition to the primary analysis, sensitivity analyses were conducted to verify the robustness of the results by varying the follow-up periods (minimum of 12 months) and excluding participants with metabolic syndrome components at baseline. Adjusted hazard ratios (AHRs) with 95% confidence intervals (CIs) were calculated to quantify the risk of metabolic syndrome associated with HTP use.

## RESULTS

Of the participants, 59.8% were male, and 40.2% were female, with mean ages of 41.1 years and 38.1 years at baseline, respectively (Table 1). Follow-up comprised 3013644 person-months, during which 16117 participants met the criteria for incident metabolic syndrome. Among the 16117 participants developing metabolic syndrome, 5866 meeting the criteria prior to completing 12 months of follow-up were excluded from the final analysis. Thus, 10251 incident cases that occurred during 2949926 person-months of follow-up were involved in the final analysis (Figure 1).

**Table 1:** Characteristics of study participants at baseline, 1 January to 31 December 2019 (N=183870)

*Characteristics*	*Men*	*Women*
	*n (%)*	*n (%)*
**Total**	110021 (59.8)	73849 (40.2)
**Age** (years), mean (SD)	41.1 (9.7)	38.1 (10.1)
**Conventional cigarettes** (CCs)		
Never smoker	42010 (38.2)	67599 (91.5)
Former smoker	32317 (29.4)	3283 (4.5)
Current smoker	35694 (32.4)	2967 (4.0)
**Electronic vaping cigarettes** (EVCs)		
Never user	94203 (85.6)	70739 (95.8)
User	15818 (14.4)	3110 (4.2)
**Heated tobacco products** (HTPs)		
**Lifetime use**		
Never user	87886 (79.9)	71833 (97.3)
User	22135 (20.1)	2016 (2.7)
**Current use**		
Never user	88156 (80.1)	71910 (97.4)
Former user	13720 (12.5)	1134 (1.5)
Current user	8145 (7.4)	805 (1.1)
**Duration of use** (years)		
Non-user	88287 (81.5)	71943 (97.8)
1	12602 (11.6)	1153 (1.6)
2	5220 (4.8)	294 (0.4)
≥3	2186 (2.0)	173 (0.2)
**Frequency per day**		
Non-user	88262 (80.7)	71935 (97.7)
1–5	5380 (4.9)	1047 (1.4)
6–10	9451 (8.7)	578 (0.8)
11–15	2930 (2.7)	61 (0.1)
≥16	3298 (3.0)	35 (0.1)
**Duration after quitting** (years)		
Non-user	88156 (81.5)	71910 (97.8)
1	5757 (5.3)	489 (0.7)
2–3	1026 (1.0)	72 (0.1)
≥4	327 (0.3)	35 (0.1)

Table 1 shows the baseline distribution of use of conventional cigarettes, EVC, and HTP among 183870 participants at baseline. Consistent with smoking patterns in Korea, the prevalence of current use of conventional cigarettes was higher among men than women (32.4% for men vs 4.0% for women). Considering the duration of HTP use, 11.6% of men and 1.6% of women had used them for one year, while 2.0% of men and 0.2% of women had used them for three or more years. The most common frequency of HTP daily use was 6–10 times per day for men (8.7%) and 1–5 times per day for women (1.4%).

**Figure 2:**
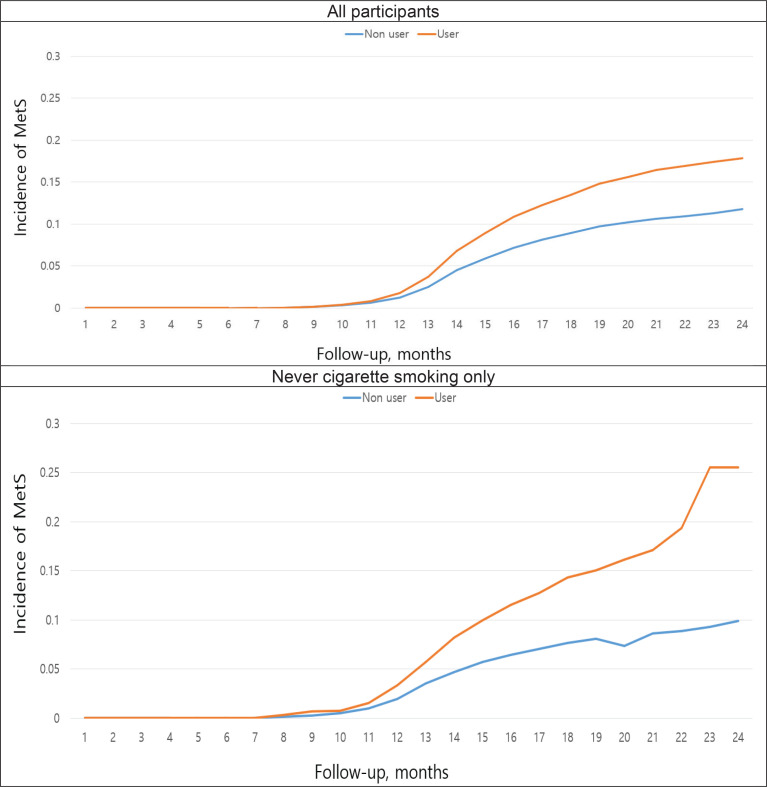
Effect of hea ted tobacco product on metabolic syndrome

Figure 2 shows the incidence of metabolic syndrome according to HTP use during up to 24 months of follow-up. Among all participants, a difference in the incidence of metabolic syndrome became apparent after 11 months of follow-up. However, among never smokers who did not smoke regular cigarettes, the difference in the occurrence of metabolic syndrome became apparent earlier, after 8 months of follow-up (Figure 2). Overall, after 24 months of follow-up, the incidence of metabolic syndrome was 2.5-fold higher for HTP users (25%) than for non-HTP users (10%).

Table 2 provides the metabolic syndrome incidence and adjusted hazard ratio by the three patterns of tobacco product use. The incidence rate of metabolic syndrome was 253.8/100000 person-months (PMs) in never smokers of conventional cigarettes. The incidence rate of metabolic syndrome among never EVC users was 336.1/100000 PMs, and the incidence rate among never HTP users was 325.7/100000 PMs, higher than the incidence rate among never smokers of conventional cigarettes (253.8/100000 PMs), likely reflecting dual use. Among those who had only one component of metabolic syndrome at baseline, the highest risk for incident metabolic syndrome was among current users of HTPs (Supplementary file Table 1). To examine the incidence of metabolic syndrome in relationship to HTP use, additional analysis was performed on never smokers of conventional cigarettes (Table 3). For users of EVCs, the incidence of metabolic syndrome was similar to that of non-users. However, for HTP users, the incidence of metabolic syndrome among non-users was 252.0/100000 PMs, whereas, for former users, it was 536.0/100000 PMs, and for current users, 427.5/100000 PMs. Supplementary file Table 2 presents the number of incident metabolic syndrome cases in 2020 stratified by the number of metabolic components in 2019 and HTP use status. The proportion of individuals with two metabolic syndrome components was consistently the highest among current HTP users in both 2019 and 2020.

**Table 2:** Incidence and hazard ratio (HR) for metabolic syndrome by use of tobacco products (N=183870)

	*Person-months (PMs)*	*Rate Per 100000 PMs*	*AHR (95% CI)*
**Conventional cigarettes** (CCs)			
Never smoker	1769985	253.8	1.0
Former smoker	561050	516.5	1.10 (1.04–1.16)
Current smoker*	618890	462.1	1.30 (1.22–1.38)
**Electronic vaping cigarettes** (EVCs)			
Non-user	2655427	336.1	1.0
User	294499	450.3	1.09 (1.02–1.17)
**Heated tobacco products** (HTPs)			
**Lifetime use**			
Non-user	2578698	325.7	1.0
Former user	234660	508.4	1.15 (1.15–1.25)
Current user	136567	481.8	1.10 (1.03–1.18)
**Never conventional cigarette smokers only**			
**Electronic vaping cigarettes** (EVCs)			
**Lifetime use**			
Non-user	1725178	253.9	1.0
User	44807	252.2	0.91 (0.75–1.10)
**Heated tobacco products** (HTPs)			
**Current use**			
Non-user	1756725	252.0	1.0
Former user	8582	536.0	1.41 (0.91–2.20)
Current user	4678	427.5	**1.68 (1.25–2.26)**

AHR: adjusted hazard ratio; adjusted for age, sex, exercise, heavy drinking, cigarette smoking status, and e-cigarette use.

* Current smokers were defined based on the World Health Organization (WHO) classification as individuals who have smoked more than 100 cigarettes in their lifetime and currently smoke.

**Table 3:** Effects of duration of heated tobacco products (HTPs) use in 2019 on metabolic syndrome in 2020, by current cigarette smoking status (N=183870)

*Duration of HTP use* *(years)*	*Total* *(10251/178004)*	*Never smoker* *(4493/106982)*	*Former smoker* *(2898/33999)*	*Current smoker* *(2860/37023)*
	*AHR (95% CI)*	*AHR (95% CI)*	*AHR (95% CI)*	*AHR (95% CI)*
**Former + current user**				
Non-user	1	1	1	1
1	1.07 (0.99–1.15)	1.36 (0.88–2.09)	0.99 (0.90–1.08)	1.04 (0.92–1.18)
2	1.20 (1.08–1.32)	1.59 (0.90–2.81)	1.16 (1.02–1.32)	1.07 (0.91–1.27)
≥3	1.10 (0.93–1.29)	**2.17 (1.31–3.62)**	1.08 (0.89–1.33)	0.84 (0.62–1.13)
**Former user**				
Non-user	1	1	1	1
1	1.12 (1.02–1.24)	1.27 (0.61–2.68)	1.06 (0.94–1.20)	1.03 (0.87–1.22)
2	1.30 (1.00–1.68)	NE	1.14 (0.74–1.76)	1.28 (0.93–1.77)
≥3	1.12 (0.73–1.72)	3.20 (1.52–6.72)	0.63 (0.20–1.94)	0.84 (0.47–1.53)
**Current user**				
1	1.03 (0.94–1.12)	1.40 (0.82–2.37)	0.94 (0.85–1.05)	1.05 (0.88–1.25)
2	1.18 (1.06–1.31)	1.72 (0.97–3.03)	1.16 (1.02–1.33)	1.02 (0.84–1.23)
≥3	1.09 (0.92–1.30)	1.69 (0.84–3.40)	1.11 (0.90–1.36)	0.84 (0.59–1.19)

AHR: adjusted hazard ratio; adjusted for age, sex, exercise, heavy drinking, cigarette smoking status, and e-cigarette use. Adjusted for age, sex, exercise, heavy drinking, conventional cigarette smoking status, and e-cigarette use. NE: not estimable

Table 3 describes the variation in incidence of metabolic syndrome by duration of HTP use. The use of HTPs and conventional cigarettes stratified participants. Among total participants (former and current HTP users), two years of use of HTPs significantly elevated the risk of metabolic syndrome by 20% (HR=1.20; 95% CI: 1.08–1.32). However, when the analysis was limited to never smokers, the associations of HTP use with the occurrence of metabolic syndrome increased, although it was not statistically significant (HR=1.59; 95% CI: 0.90–2.81). Among former and current HTP users who used them for three years or longer without smoking conventional cigarettes, the risk of metabolic syndrome was 2.17-fold higher than among non-HTP users. Former HTP users of ≥3 years who never smoked conventional cigarettes showed a 3.20-fold higher risk of metabolic syndrome compared to non-HTP users. Table 4 provides the effect of daily use frequency of HTP use on the risk of metabolic syndrome. Among total participants (former and current HTP users), those who used HTPs more than 16 times a day showed a 1.33-fold higher risk of metabolic syndrome compared to those who used HTPs 1–5 times a day.

**Table 4:** Effects of frequency of heated tobacco products (HTPs) use in 2019 on metabolic syndrome in 2020, by current cigarette smoking status (N=183870)

*Frequency of use per* *day*	*Total* *(10251/178004)*	*Never smoker* *(4493/106982)*	*Former smoker* *(2898/33999)*	*Current smoker* *(2860/37023)*
	*AHR (95% CI)*	*AHR (95% CI)*	*AHR (95% CI)*	*AHR (95% CI)*
**Former + current user**				
Non-user	1.0	1.0	1.0	1.0
1–5	1.01 (0.91–1.12)	1.28 (0.75–2.16)	0.98 (0.87–1.11)	0.86 (0.70–1.07)
6–10	1.08 (1.00–1.17)	1.92 (1.34–2.74)	1.03 (0.93–1.14)	0.92 (0.80–1.07)
11–15	1.15 (1.01–1.31)	1.62 (0.84–3.12)	1.05 (0.89–1.24)	1.15 (0.93–1.42)
≥16	1.33 (1.18–1.49)	1.70 (0.94–3.07)	1.26 (1.09–1.45)	1.35 (1.11–1.64)
**Former user**				
Non-user	1.0	1.0	1.0	1.0
1–5	1.08 (0.92–1.26)	1.80 (0.97–3.35)	0.96 (0.78–1.19)	1.01 (0.80–1.29)
6–10	1.13 (0.99–1.28)	0.82 (0.26–2.54)	1.14 (0.97–1.35)	0.91 (0.73–1.13)
11–15	1.19 (0.93–1.53)	4.23 (1.75–10.19)	1.19 (0.88–1.62)	0.85 (0.53–1.35)
≥16	1.26 (1.04–1.53)	0.55 (0.08–3.92)	1.13 (0.88–1.47)	1.37 (1.02–1.83)
**Current user**				
1–5	0.97 (0.85–1.11)	0.74 (0.28–1.98)	0.99 (0.86–1.14)	0.60 (0.39–0.90)
6–10	1.06 (0.96–1.16)	2.24 (1.54–3.26)	0.99 (0.88–1.11)	0.93 (0.78–1.11)
11–15	1.14 (0.98–1.32)	0.92 (0.34–2.45)	1.01 (0.83–1.22)	1.26 (0.99–1.60)
≥16	1.36 (1.19–1.56)	2.14 (1.15–3.99)	1.32 (1.11–1.56)	1.33 (1.03–1.71)

AHR: adjusted hazard ratio; adjusted for age, sex, exercise, heavy drinking, cigarette smoking status, and e-cigarette use.

## DISCUSSION

We conducted a prospective cohort study comprising 178004 adults who visited the Korea Medical Research Institute for a general health checkup in 2019 and returned during 2020. In this cohort, we found that the use of HTP tobacco products increased the risk of incident metabolic syndrome, and that increase extended across groups of mixed use of tobacco products, including conventional cigarettes and EVCs. The HTP products have been on the market for only a few years, so evidence of their risks to health remains quite limited. However, the extensive research on conventional cigarettes and emerging research on EVCs provides a starting point for considering their potential risks. Overall, a large body of research suggests the potential for HTPs to have cardiovascular and metabolic effects that could increase the risk for metabolic syndrome. Our findings provide early evidence for an increased risk of metabolic syndrome associated with the use of HTP products.

There is little prior research on HTPs compared with our study’s findings. Previous studies have found that HTPs emit lower levels of certain harmful chemicals compared to combustible cigarettes, yet release carcinogenic and toxic compounds, such as carbonyls and free radicals, which contribute to long-term health risks^22,23^. For active smoking, epidemiological studies have shown a strong association with metabolic syndrome. A 2012 systematic review and meta-analysis that included 13 prospective cohort studies with 56691 participants found a significant positive association between active smoking and risk of metabolic syndrome (pooled relative risk, RR=1.26; 95% CI: 1.10–1.44)^24^. For EVCs, a Korean nationwide population-based study using cross-sectional data from the period 2013–2015 (n=14738) found that their use was significantly associated with components of metabolic syndrome, including abdominal obesity, high triglycerides, and high fasting blood glucose^18^. With adjustment for conventional cigarettes and other potential confounding variables, current EVC usage was associated with increased risk for metabolic syndrome (OR=1.40; 95% CI: 1.08–1.81). Among active smokers, EVC use was not significantly associated with metabolic syndrome (OR=1.13; 95% CI: 0.82–1.55). We could not identify other studies that directly addressed HTPs and the risk for metabolic syndrome.

Tobacco smoking has been causally linked to type 2 diabetes mellitus and shown to have effects on metabolism^25^. The patterns of association of smoking with obesity and blood pressure are complex and not deemed to be causal. Of the components in the emissions from HTP devices, nicotine is the most probable contributor to the increased risk for metabolic syndrome. It affects insulin release and promotes insulin resistance in people with type 2 diabetes. Nicotine affects metabolism, including lipolysis, via activation of the sympathetic nervous system. Inhaled HTP emissions may also promote inflammation, further enhancing the risk for metabolic syndrome.

Thus, our findings are plausible, but the strength of the association among never smokers of cigarettes is surprising (Table 2). While the follow-up time of never cigarette but current HTP users was relatively small (n=4678 person-months), the HR was significantly elevated and showed an almost 70% increased risk. This is one of the most informative findings of the analyses as there is little potential for confounding by smoking. The finding needs replication but indicates a need for caution in assuming that HTP products may pose a lower risk than cigarettes for all health outcomes. The findings of a 2020 systematic review indicate lower emissions than from conventional cigarettes^8^. However, in one model of vascular endothelial function, aerosol from an IQOS device had the same effect as cigarette smoke^9^.

### Limitations

In interpreting the findings, certain study limitations need to be considered. First, the follow-up period is relatively short, which may be insufficient to capture the long-term effects of heated tobacco product (HTP) use on the development of metabolic syndrome. Extending the follow-up period to 5 or 10 years could provide a more comprehensive understanding of the long-term metabolic risks, as metabolic syndrome and its components, such as obesity and hypertension, often develop over an extended period. Second, there is potential for residual confounding. Although we adjusted for several key confounders, such as age, sex, smoking status, and alcohol consumption, there may be other unmeasured factors influencing the relationship between HTP use and metabolic syndrome. For example, dietary habits, stress levels, or genetic predispositions could have affected the outcomes.

There is also a potential for selection bias as smokers who developed one or two components of metabolic syndrome may begin to switch to HTP devices from cigarettes, seeking a lower risk way to manage their nicotine addiction. Third, information bias is another limitation, as the study relied on participants’ self-reported use of tobacco products. This could introduce reporting errors, particularly underreporting or misclassification of tobacco use. To mitigate this, future studies should consider incorporating objective biomarkers of nicotine exposure, such as cotinine levels in blood or urine, to provide a more accurate assessment of tobacco use and minimize the risk of reporting bias. Additionally, the study did not fully address the complex patterns of dual or multiple tobacco product use (e.g. HTP use combined with conventional cigarettes or electronic vaping cigarettes). These patterns could have synergistic or independent effects on metabolic syndrome risk, and future analyses should explore these combinations to provide a clearer understanding of the associated risks.

Lastly, gender-specific analyses were not performed, even though there is evidence that metabolic syndrome risk may differ between men and women due to hormonal and biological differences. Including gender-specific analyses could reveal important distinctions in vulnerability to metabolic syndrome among different user groups and offer insights into how public health policies should be tailored for men and women.

The medical record information used to determine the presence of metabolic syndrome was comprehensive, ensuring the validity of the outcomes. However, the generalizability of these findings to populations outside Korea remains uncertain and should be tested in broader geographical and cultural contexts.

## CONCLUSIONS

Our findings suggest a possible increased risk for metabolic syndrome among users of HTP devices, although further research is needed to confirm these results. The observed associations highlight the importance of continued monitoring of HTP use and its health effects, particularly given the evolving landscape of tobacco products. Additional studies are required to fully assess the long-term consequences and clarify potential limitations due to proxy measures.

## Supplementary Material



## Data Availability

The data supporting this research are available from the authors on reasonable request.
