# Supplementary material for: The effect of heated tobacco products on metabolic syndrome: A cohort study
Source: Tob Induc Dis. 2024 Dec 16;22:10.18332/tid/194490. doi: 10.18332/tid/194490 (PMC11647454; doi:10.18332/tid/194490)
Supplement: Supplementary file 1 [file TID-22-187-s1.pdf]

Supplementary Material 1. Measurement of tobacco product use from Korean National Institute (KMI) health examination survey

[Conventional cigarette]

[4] Have you smoked more than 5 packs of conventional cigarette in your lifetime?

① No -> Move to [5]

② Yes, but currently quitted -> Move to [4-1]

③ Yes, currently smoking-> Move to [4-2]

[4-1] If you quitted smoking conventional cigarette

- How long did you smoke: ________ years
- How many cigarettes did you smoke per day on average before quitting? : ________ cigarettes
- How long has it been since you quit smoking: ________ years

[4-2] If you still smoke conventional cigarette

- How long did you smoke: ________ years
- How many cigarettes did you smoke per day on average? : ________ cigarettes

[of Heated tobacco products (HTP)]

[5] Have you ever used of Heated tobacco products (HTP)?

① No -> Move to [6]

② Yes-> Move to [5-1]

[5-1] Do you currently use of Heated tobacco products (HTP)during last month?

① No ② 1-2 day per month ③ 3-9 day per month

④ 10-29 day per month ⑤ Everyday

[Electronic vaping cigarettes (EVC)]

[6] Have you ever used electronic vaping cigarettes?

① No

② Yes -> Move to [6-1]

[6-1] Have you ever used electronic vaping cigarettes during last month

③ No ② 1-2 day per month ③ 3-9 day per month

④ 10-29 day per month ⑤ Everyday

Supplementary Material 2. Change of metabolic components between 2019 and 2020 stratified by HTP use status (n=178,004)

n (%)	Number of components in 2020			
Number of components in 2019	0	1	2	3>
	among non HTP users (n=82,808)			
0	47,883 (91.71)	20,280 (87.66)	5,395 (84.52)	862 (82.88)
1	2,636 (5.05)	1,742 (7.53)	610 (9.56)	110 (10.58)
2	1,691 (3.24)	1,113 (4.81)	378 (5.92)	68 (6.54)
	among former HTP users (n=61,016)			
0	13,861 (88.14)	23,909 (86.56)	11,997 (83.91)	2,780 (82.52)
1	1,156 (7.35)	2,339 (8.47)	1,507 (27.89)	402 (11.93)
2	710 (4.51)	1,374 (4.97)	794 (5.55)	187 (5.55)
	among current HTP users (n=34,180)			
0	2,804 (85.18)	8,724 (83.70)	12,020 (81.98)	4718 (81.32)
1	296 (8.99)	1,090 (10.46)	1,674 (11.42)	681 (11.7)
2	192 (5.83)	609 (5.84)	969 (6.61)	403 (6.95)

Supplementary Table 1. Effects of heat-not-burn in 2019 on metabolic syndrome in 2020, according to number of metabolic syndrome components in 2019 (N=183,870)

Number of metabolic syndrome components in 2019	Heat-not-burn	Person month (PM)	Rate /100,000 PM	Adjusted HR (95% CI)
0	Experience			
	No user	1,244,031	72.5	1.0
	Former user	85,192	129.1	1.08 (0.83-1.41)
	Current user	53,107	128.0	1.01 (0.81-1.26)
1	Experience			
	No user	873,776	318.2	1.0
	Former user	89,168	450.8	1.01 (0.86-1.18)
	Current user	49,353	378.9	**1.11 (0.99-1.25)**
2	Experience			
	No user	460,891	1,023.7	1.0
	Former user	60,300	1,129.4	**1.16 (1.04-1.29)**

	Current user	34,106	1,181.6	1.03 (0.94-1.13)
Total	Experience			
	No user	2,578,698	325.7	1.0
	Former user	234,660	508.4	**1.15 (1.15-1.25)**
	Current user	136,567	481.8	**1.10 (1.03-1.18)**

Supplementary Table 2. Number of incident metabolic syndrome cases in 2020 according to number of metabolic components in 2019 among stratified by HTP use status (n=178,004)

n (%)<br>Number of components in 2019	Metabolic syndrome status in 2020<br>No Metabolic syndrome	Yes Metabolic syndrome
	among non HTP users (n=82,808)	
0	73,558 (90.00)	902 (83.52)
1	4,988 (6.10)	110 (10.19)
2	3,182 (3.89)	68 (6.30)
	among former HTP users (n=61,016)	
0	49,767 (86.33)	2,780 (82.52)
1	5,002 (8.68)	402 (11.93)
2	2,878 (4.99)	137 (5.55)
	among current HTP users (n=34,180)	
0	23,548 (82.98)	4,718 (81.32)
1	3,060 (10.78)	681 (11.74)
2	1,770 (6.24)	403 (6.95)

© 2024 Jee Y. et al.
